# A4 HELMINTH-DERIVED METABOLITES INDUCE A TOLEROGENIC PROFILE IN DENDRITIC CELLS AND ALLEVIATE EXPERIMENTAL COLITIS

**DOI:** 10.1093/jcag/gwab049.003

**Published:** 2022-02-21

**Authors:** N O Malacco, E A Siciliani, A G Madrigal, I Cestari, A Jardim, M Stevenson, F Lopes

**Affiliations:** Institute of Parasitology, McGill University, Montreal, QC, Canada

## Abstract

**Background:**

Inflammatory bowel diseases (IBD) are chronic inflammatory diseases characterized by abdominal pain, bloody diarrhea, fatigue, weight loss, and diminished quality of life. The morbidity associated with IBD is a result of loss of tolerance towards the gastrointestinal commensal microbiota. The high incidence of IBD in Western societies is inversely correlated with the low incidence of intestinal helminth parasite infections, potentially due to the ability of the helminth parasite to induce tolerance by inducing tolerogenic dendritic cells (tolDC) polarization. Although macromolecules from helminth such as proteins and polysaccharides have been shown to polarize tolDCs, to characterize a novel pathway, in this study we focused on small molecules such as metabolites in the helminth secretome. We hypothesized that helminth-derived metabolites (HDMs) polarize DCs towards a tolerogenic phenotype, which alleviates colitis.

**Aims:**

To evaluate and characterize the tolerogenic response induced by HDMs in DCs, and its ability to alleviate colitis.

**Methods:**

*Heligmosomoides polygyrus* worms were culture for 24 h and HDMs were isolated from conditioned media by chromatography. Bone marrow dendritic cells (BMDCs) were differentiated with GM-CSF for 8 days and then incubated with HDM for 4 h before LPS stimulation for 20 h. Cytokine secretion was measured by ELISA. The transcriptome of DCs treated with HDMs was assessed by RNAseq. Colitis was induced by giving 3% DSS in drinking water for 5 days followed by 3 days of tap water. The anti-colitic effect of HDMs was assessed by daily treatment with HDM or DCs treated with HDM in the 3 days of tap water.

**Results:**

Pre-treatment with HDM decreased LPS-induced TNF and increased IL-10 release by BMDCs, compared to control BMDCs. Colitic mice treated with HDM presented lower disease activity scores, less colon shortening, decreased weight loss, and healthier histopathology compared to vehicle-treated colitic mice. Importantly, there was an increased frequency of CD11c+ CD103+ DCs in the colon of HDM-treated mice, suggesting that HDM alleviates colitis by increasing the abundance of tolDCs in the colon. Adoptive transfer of HDM-treated DCs also reduced the severity of colitis compared with vehicle-treated mice or mice that received naïve DCs. These results indicate that HDM induced tolerogenic DCs, which in turn ameliorates DSS colitis. RNAseq showed that HDM upregulated 183 and downregulated 76 genes. These differentially expressed genes may indicate a novel mechanism by which helminths induce a tolerogenic profile in DCs.

**Conclusions:**

HDMs induce tolerogenic DCs and alleviate DSS-induced colitis.

**Funding Agencies:**

NSERC and FRQNT

